# An accelerated dose escalation with a grass pollen allergoid is safe and well-tolerated: a randomized open label phase II trial

**DOI:** 10.1186/s13601-016-0093-z

**Published:** 2016-02-02

**Authors:** A. M. Chaker, B. Al-Kadah, U. Luther, U. Neumann, M. Wagenmann

**Affiliations:** 1 Department of Otolaryngology and ZAUM, Klinikum rechts der Isar, Technische Universität and Helmholtz Center Munich, Ismaninger Straße 22, 81675 Munich, Germany; 2 Department of Otorhinolaryngology, Saarland University Medical Centre, Kirrberger Straße, 66421 Homburg, Saar, Germany; 3 Joint Practice of Dermatologists, Allergology - Phlebology, Kaiser-Joseph-Str. 145, 79098 Freiburg im Breisgau, Germany; 4 ENT Medicine, Bahnhofstr. 18, 39326 Wolmirstedt, Germany; 5 Department of Otorhinolaryngology, University Hospital Düsseldorf, Moorenstr. 5, 40225 Düsseldorf, Germany

**Keywords:** Accelerated dose escalation, Allergoid, Safety, Subcutaneous immunotherapy, Tolerability

## Abstract

**Background:**

The number of injections in the dose escalation of subcutaneous immunotherapy (SCIT) is small for some currently used hypoallergenic allergoids, but can still be inconvenient to patients and can impair compliance. The aim of this trial was to compare safety and tolerability of an accelerated to the conventional dose escalation scheme of a grass pollen allergoid.

**Methods:**

In an open label phase II trial, 122 patients were 1:1 randomized for SCIT using a grass pollen allergoid with an accelerated dose escalation comprising only 4 weekly injections (Group I) or a conventional dose escalation including 7 weekly injections (Group II). Safety determination included the occurrence of local and systemic adverse events. Tolerability was assessed by patients and physicians.

**Results:**

Treatment-related adverse events were observed in 22 (36.1 %) patients in Group I and 15 (24.6 %) in Group II. Local reactions were reported by 18 patients in Group I and 11 in Group II. Five Grade 1 systemic reactions (WAO classification) were observed in Group I and 2 in Group II. Grade 2 reactions occurred 3 times in Group I and 2 times in Group II. Tolerability was rated as “good” or “very good” by 53 (86.9 %) patients in Group I and 59 (100 %) in Group II by investigators. Forty-eight patients in Group I (80.0 %) and 54 in Group II (91.5 %) rated tolerability as “good” or “very good”.

**Conclusions:**

The dose escalation of a grass pollen allergoid can be accelerated with safety and tolerability profiles comparable to the conventional dose escalation.

## Background

Sensitization to grass pollen allergens is the most prevalent cause of respiratory allergy in Europe [1]. For more than 100 years, grass pollen allergy has been treated with subcutaneous immunotherapy (SCIT) applying increasing allergen doses to reach the efficacious maintenance phase. SCIT was demonstrated to be effective in both allergic asthma [2] and allergic rhinitis [3]. Like all long-term treatments [4], adherence to SCIT is affected by a number of factors. One of the most relevant factors is inconvenience related to commuting to receive the allergy injections [5–7]. Accelerated dose escalation allows patients to reach effective doses faster [8, 9], while increasing the adherence to treatment [10, 11]. The shift from aqueous extracts to depot preparations, adsorbed to aluminium hydroxide or other adjuvants, allows the number of injections to be reduced significantly. Cluster and rush schemes were introduced with the aim of further accelerating the dose escalation. However, these schemes carry an increased risk of severe systemic reactions [8, 12]. The development of chemically modified allergens (so-called high-dose hypoallergenic preparations or allergoids) achieved the combined goal of accelerating dose escalation and administering therapeutic doses with a reduced potential for side effects while maintaining immunogenicity [13, 14]. Grass pollen allergoids have been demonstrated to be effective and safe when seven preseasonal injections were used to reach the maintenance dose [15]. Accelerated dose escalation schemes with different allergoid preparations have also been demonstrated to be safe and effective in previous studies [17, 18]. The purpose of this trial was to evaluate the safety and tolerability of an accelerated dose escalation scheme for a grass pollen allergoid, allowing the recommended maintenance dose to be reached within a time span of 3 weeks only. This accelerated dose escalation scheme can reduce the inconvenience to patients and improve the attractiveness of SCIT.

## Methods

This was a randomized open label phase II multicentre clinical trial performed in Germany. Patients were randomized to 2 parallel active treatment groups in 1:1 ratio. Group I received an accelerated dose escalation scheme of 4 injections at weekly intervals, and Group II received the conventional dose escalation scheme with 7 weekly injections.

### Inclusion criteria

Male and female outpatients aged 18–65 years with grass pollen-induced IgE-mediated seasonal allergic rhinoconjunctivitis with or without bronchial asthma confirmed by a positive skin prick test (grass pollen wheal size ≥3 mm in diameter) and a grass pollen specific IgE level of ≥0.70 kU/L, were included. Patients also had to have their major discomfort during the grass pollen season. Diagnosed asthma, if present, had to be classified as “controlled” according to the GINA guidelines [19].

### Test product

Allergovit^®^ 6-grasses (Allergopharma GmbH & Co. KG, Reinbek, Germany), a 100 % mixture of allergens from 6 grass pollen species (*Holcus lanatus, Dactylis glomerata, Lolium perenne, Phleum pratense, Poa pratensis, and Festuca pratensis*) chemically modified with formaldehyde to produce an allergoid, which is then co-precipitated with aluminium hydroxide, was used. Allergovit^®^ 6-grasses is specified in therapeutic units per mL (TU/mL), provided in two strengths (A: 1000 TU/mL and B: 10,000 TU/mL).

### Treatment

**Fig. 1:**
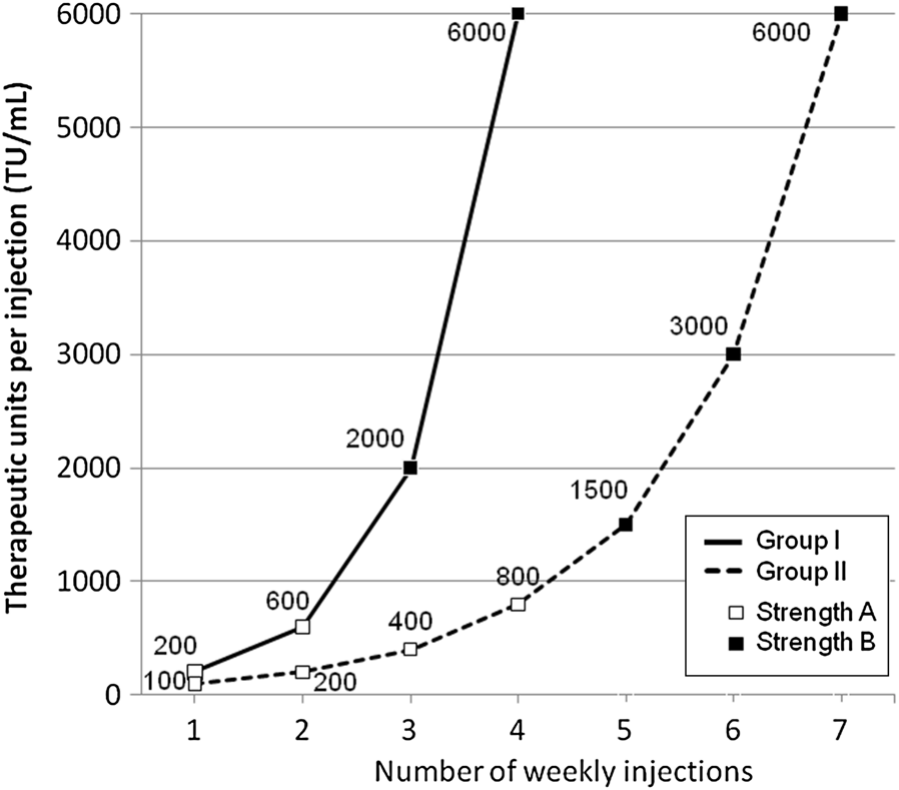
Dose escalation schemes in Group I and II. The maximal dose of 6000 TU was repeated after 14 and 28 days in both groups

Patients randomized to Group I were treated for up to 12 weeks, and patients randomized to Group II were treated for up to 15 weeks. The injections of gradually increasing doses were administered weekly, and the doses were increased one step at a time, provided the previous dose had been well tolerated. In Group I, patients received 2 injections of strength A and 2 injections of strength B, with volumes of 0.2 and 0.6 mL, respectively. In Group II, patients received 4 injections of strength A with volumes of 0.1, 0.2, 0.4 and 0.8 mL, and 3 injections of strength B with volumes of 0.15, 0.3 and 0.6 mL. When the maximum dose had been reached, both groups received maintenance treatment with 2 maximum dose injections of 6000 TU/ml after 14 and 28 days. This dose corresponds to a content of 25 μg of grass group 5 allergens [20]. To enable rapid intervention in case of allergic reactions, patients in Group I received a venous catheter prior to each injection remaining in place until the end of supervision. The minimum supervision time at the trial site was 120 min for Group I and 30 min for Group II. The 2 treatment schemes are shown in Fig. 1.

### Criteria for evaluation

Adverse events (AEs) and serious adverse events (SAEs) were assessed by the investigator and coded using MedDRA version 15.0 according to primary System Organ Class (SOC) and Preferred Term (PT). The safety endpoints were as follows:

- AEs considered to be related to the trial medication by the investigator;
- intensity of treatment-emergent AEs (TEAEs) as judged by the investigator (mild = transient symptoms, no interference with the patient’s daily activities; moderate = marked symptoms, moderate interference with the patient’s daily activities; severe = considerable interference with the patient’s daily activities);
- incidence and intensity of systemic reactions after injections according to a modified WAO classification using only the symptoms (no consideration of epinephrine administration) documented and judged by the investigator [21]. The classification was supervised by the Data Safety Monitoring Board;
- number, incidence, type, and intensity of local AEs (local reactions at the injection site >5 cm);
- number of local reactions at the injection site ≤5 cm, which were not considered as AEs;
- changes in laboratory values (haematology, clinical chemistry, and urinalysis) measured before and after the treatment phase;
- changes in vital signs and lung function measured before, during, and after treatment.
- the assessment of the overall tolerability of treatment by the investigator and the patient was performed using a 5-point Likert scale (1 = very bad and 5 = very good) [22].

### Statistical methods

Safety data were analysed descriptively. Due to the exploratory design of this study, there was no formal estimation of sample size to account for type I error rate, power, standard deviation, and effect size. However, a sample size of 120 patients, 60 patients per group, was chosen, and the patients were randomized to the 2 treatment groups. This sample size was considered sufficient to guarantee a probability of 95 % that AEs with a true incidence rate of 5 % in one treatment group occur at least once in that treatment group. Thus, the sample size used in this trial was considered to allow for the observation of less frequently occurring AEs and the comparison of AE profiles and changes in vital signs and laboratory values.

### Ethical conduct of the trial

Patients willing to participate in the trial were asked to provide written informed consent after being given sufficient time to consider participation and the opportunity to ask any questions they had regarding the study. The informed consent form was signed and personally dated by both the patient and the investigator. The trial was conducted in accordance with the trial protocol, the International Conference on Harmonization guideline for Good Clinical Practice, applicable local regulations and the Declaration of Helsinki.

## Results

### Study population

**Fig. 2:**
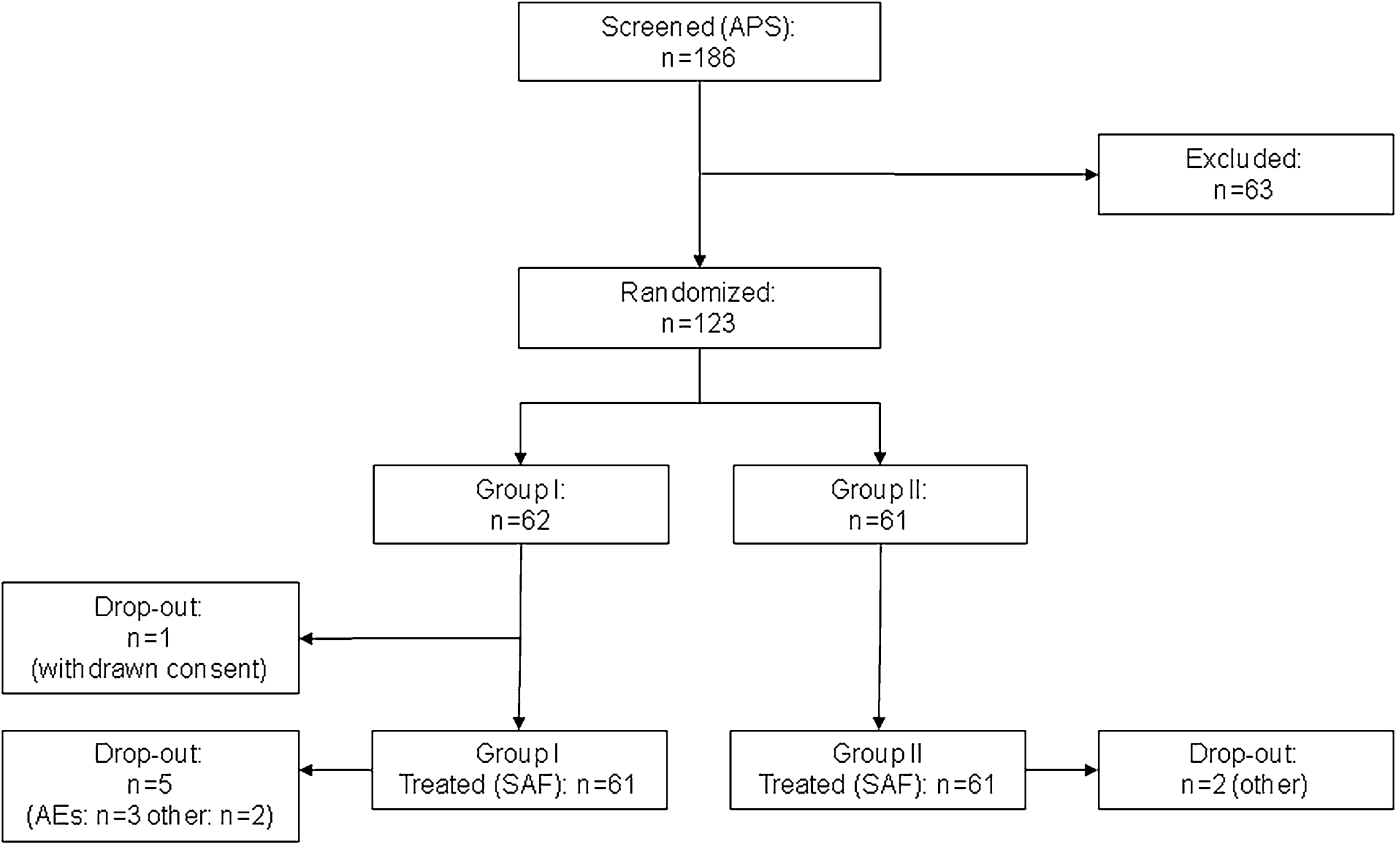
Patient flow-chart

For this study, 186 patients (All-Patients Set—APS) were screened and 123 were randomized. One patient withdrew his consent prior to the first drug administration. One-hundred twenty-two patients, 61 in each treatment group, received at least one dose of trial medication (Safety Set—SAF). In the Safety Set 7 patients left the study prematurely, including 5 Group I patients and 2 Group II patients. The reasons for premature trial termination in Group I were occurrence of AEs (3 patients) and other reasons (2 patients). The 2 patients in Group II terminated the study due to other reasons (Fig. 2). Both treatment groups were well-balanced in their demographic and other baseline characteristics. The mean treatment durations were 68.8 ± 18.7 days and 92.3 ± 12.4 days in Groups I and II, respectively. In Groups I and II, 75.4 and 88.5 % of patients reached the maintenance dose without back dosing, respectively.

### Adverse events

Sixteen non-treatment-emergent AEs in 15 patients were reported. One non-treatment-emergent adverse event (phobia) in 1 patient in Group I was related to the trial procedure. This patient withdrew from the study before receiving any injections. During the entire trial duration, which included the dose escalation phase and 2 maintenance dose injections, 78 (63.9 %) patients reported 197 TEAEs. The occurrence of TEAEs was similar between the 2 groups. The majority (53, 43.4 %) of patients only reported mild TEAEs. One serious and severe TEAE (peritonsillar abscess), which was not related to the trial medication or procedure, was reported during the dose escalation phase in a patient in Group II receiving 0.1 mL of strength A. The most frequently reported TEAEs belonged to the SOC of “infections and infestations”. Slightly more patients in Group II reported these types of AEs (Group I: 36.1 %, Group II: 45.9 %). Eighty-one TEAEs reported by 37 (30.3 %) patients were related to the trial medication. In Group I, 22 patients (36.1 %) reported treatment-related TEAEs, compared to 15 (24.6 %) patients in Group II. Most events reported were mild in intensity (Table 1). For TEAEs related to the trial medication, the most frequent reported SOC in both Group I (29.5 % of patients) and Group II (19.7 % of patients) was “general disorders and administration site conditions” (PTs: injection site swelling, injection site erythema, injection site pruritus, injection site warmth, injection site discomfort, injection site reaction, and injection site urticaria).

**Table 1:** Intensity of systemic and local reactions

	Intensity^a^	Group I accelerated (N = 61)	Group II conventional (N = 61)
Systemic reaction	Mild	8 (13.1 %)	2 (3.3 %)
	Moderate	0 (0.0 %)	2 (3.3 %)
	Severe	0 (0.0 %)	0 (0.0 %)
Injection site swelling	Mild	10 (16.4 %)	5 (8.2 %)
	Moderate	2 (3.3 %)	1 (1.6 %)
	Severe	1 (1.6 %)	0 (0.0 %)
Injection site erythema	Mild	10 (16.4 %)	7 (11.5 %)
	Moderate	1 (1.6 %)	0 (0.0 %)
	Severe	0 (0.0 %)	0 (0.0 %)

^a^Each patient counted once under the highest intensity

### Local reactions

Thirty-eight local reactions (>5 cm) related to treatment were reported in 18 (29.5 %) patients in Group I and 27 local reactions were reported in 11 (18.0 %) patients in Group II (95 %-CI for risk difference: −3.9 % to 26.6 %). The majority of local reactions (20 events in 11 patients [18.0 %] in Group I and 17 events in 6 patients [9.8 %] in Group II) were observed under strength A treatment. During the entire study, the mean maximal diameter of local reactions per visit was highly comparable between both treatment groups, with an average maximal size of 2.1 ± 1.6 cm in Group I and 2.0 ± 1.7 cm in Group II.

### Systemic reactions

Overall, systemic allergic reactions were more frequently reported in Group I than in Group II (Group I: 8, 13.1 % vs Group II: 4, 6.6 % [CI for risk difference: −4.8–18.4 %]). Most of these reactions were WAO Grade 1. Overall, 5 reactions were Grade 2 (3 in Group I and 2 in Group II) and no reaction was classified as Grade 3, 4, or 5 (Table 2). Epinephrine was never administered.

**Table 2:** WAO classification of treatment-related systemic reactions

	Group I accelerated	Group II conventional
Reactions per number of injections		
Total number of injections	371	557
Injections without reactions	362 (97.6 %)	553 (99.3 %)
WAO Grade 1	5 (1.3 %)	2 (0.4 %)
WAO Grade 2	3 (0.8 %)	2 (0.4 %)
WAO Grade not classified	1 (0.3 %)^a^	0 (0.0 %)
Patients with systemic reactions		
Total number of patients	61	61
Patients without reaction	52 (85.2 %)	57 (93.4 %)
WAO Grade 1	5 (8.2 %)	2 (3.3 %)
WAO Grade 2	3 (4.9 %)	2 (3.3 %)
WAO Grade not classified	1 (1.6 %)^a^	0 (0.0 %)

^a^Peak expiratory flow decrease without clinical symptoms

### Laboratory parameters, vital signs, and lung function

Except for 1 patient in Group II with an abnormal platelet count at the final visit, no clinically significant change from baseline for any laboratory parameter was reported. This change was not considered an AE or related to study treatment. The lung function tests and vital sign measurements performed after the injections did not show systematic changes in any parameter for any treatment group. Vital signs at the final visit did not show clinically significant changes from baseline.

### Tolerability

**Fig. 3:**
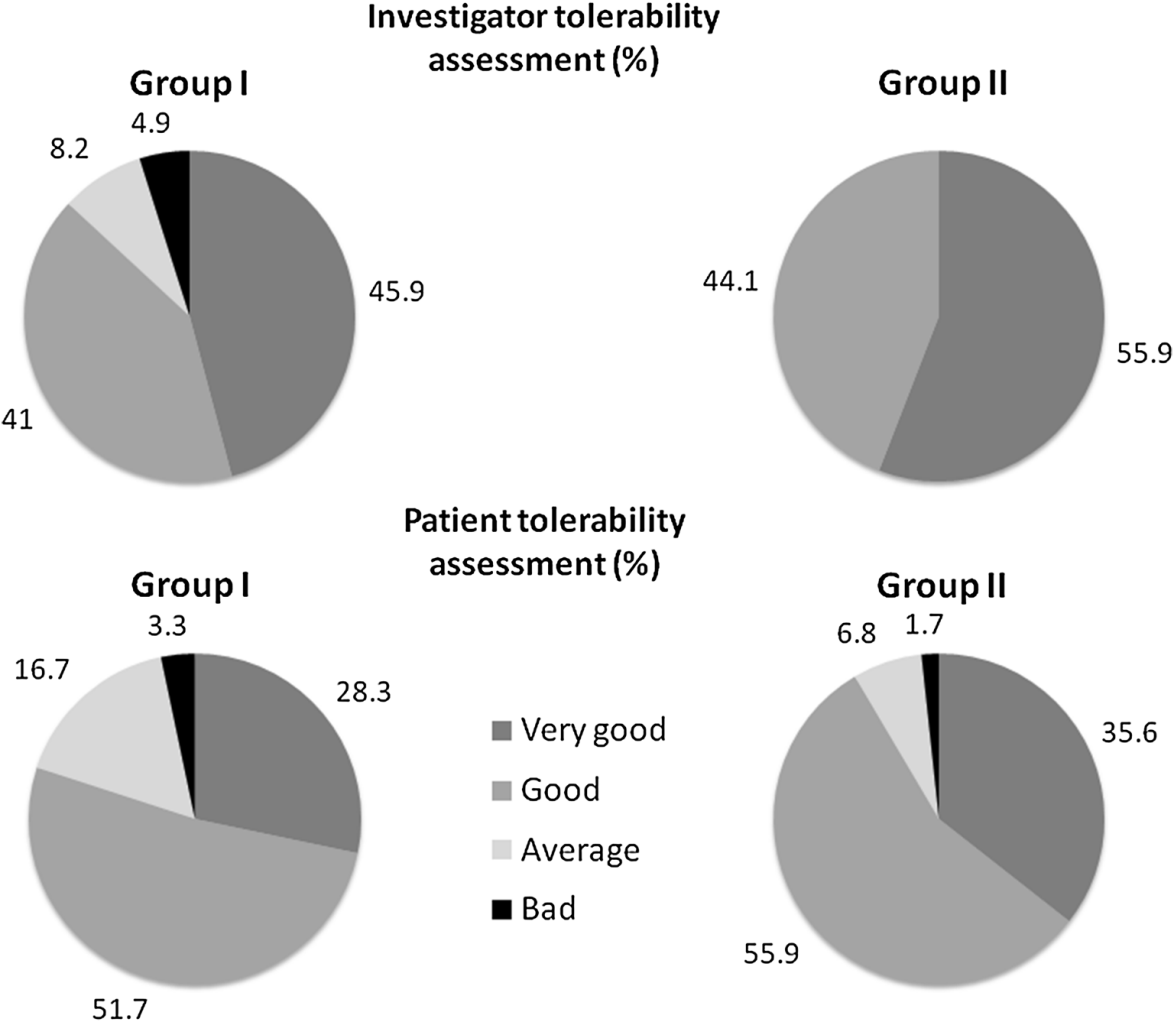
Tolerability assessment by investigators and patients

After treatment, the overall tolerability was assessed by investigators and patients separately. Investigators rated the treatment tolerability as “good” or “very good” for 53 (86.9 %) patients in Group I and 59 (100 %) patients in Group II. In Group I, 48 patients (80.0 %) and in Group II 54 patients (91.5 %) rated the treatment tolerability as “good” or “very good” (Fig. 3).

## Discussion

This trial demonstrates that the dose escalation of a high-dose grass pollen allergoid for SCIT can be accelerated to 4 injections in weekly intervals with acceptable safety and good tolerability profiles, overall comparable to those of the conventional dose escalation scheme comprising 7 injections. As expected we observed an increase of systemic events during the accelerated dose escalation corresponding to the high amount of major allergen in the vaccine. However, type and intensity of AEs, drug-related AEs, and systemic reactions were similar between the patients receiving SCIT in the accelerated dose escalation and the patients treated in the conventional scheme. An accelerated dose escalation phase offers several advantages. First of all, it allows the patients to reach the recommended maintenance dose faster. Clinical benefits and immunological responses of SCIT have been shown to appear very shortly after the maintenance dose has been reached [8, 9, 11, 23, 24]. Accelerating the dose escalation phase also addresses the issue of adherence to allergen immunotherapy (AIT). It is known from several studies, that the efficacy of SCIT, as with all long-term treatments, can be impaired by poor compliance, even if it can be argued that it is better than in sublingual AIT [4–6, 25, 26]. Lack of compliance is a restraint against AIT [27, 28]. Interestingly, in one study comparing the adherence to rush and conventional dose escalation schemes, the majority of patients who terminated were found in the conventional dose escalation group [11]. Accelerating the dose escalation phase to a duration of 3 weeks, as done in this trial, could reduce inconveniences and increase treatment adherence. Several studies with this aim have evaluated the safety of accelerated dose escalation phases for aeroallergen extracts, either by reducing the number of injections or by adopting rush or cluster schemes. Rush schemes seem to carry a higher risk of systemic reactions; in comparison, cluster schemes are characterized by a better benefit/risk ratio [8–10, 24]. In this study, we were able to show that an acceleration of the dose escalation was safe and well-tolerated, though we saw a higher frequency in local reactions and mild systemic events.

The majority of patients and investigators rated the accelerated scheme tolerability as “good” or “very good”. Some differences in tolerability can also be explained by the open label study design: for safety reasons patients in Group I had to wait 120 min after the injection, and the Federal Institute for Vaccines and Biomedicines (Paul-Ehrlich-Institute) requested that all patients in the accelerated dose escalation group were asked to carry a single use indwelling catheter with them during each injection. Therefore, patients in the accelerated dose escalation group may have experienced more psychological stress prior to and after allergen injection than patients treated with the conventional scheme. Nevertheless, the tolerability rating by the patients was positive in both schemes. The difference in the tolerability rating by the investigators may also be explained by the open study design and the additional efforts imposed on the investigators.

Meanwhile, regulatory authorities in Germany have granted marketing authorization for the accelerated updosing scheme of the investigated study drug. Efficacy of the study drug has been shown in double-blind, placebo controlled trials [15, 16], where in the latter trial a cumulative dose of 12,000 BU per protocol resulted in clinical benefit after one pre-seasonal updosing course. Patients during this study received per protocol a cumulative dose of 20.800 TU (group I) and 24.000 TU (group II), including 2 additional maintenance doses after updosing, respectively.

The efficacy of AIT is known to be dose-dependent at maintenance-dose level [29]. Examination of modified updosing schemes did not alter the induction of systemic IgG4 responses in a recent study [30].

## Conclusions

In conclusion, the dose escalation scheme of a grass pollen allergoid can be accelerated from the conventional 7–4 injections in weekly intervals with comparable safety and tolerability profiles. This accelerated dose escalation is expected to attract more patients to undergo SCIT and benefit from its clinical and immunological effects.

## Abbreviations

AE: adverse event
AIT: allergen immunotherapy
APS: All-Patients Set
GINA: Global Initiative for Asthma
MedRA: Medical Dictionary for Regulatory Activities
PT: Preferred Term
SAE: serious adverse events
SAF: Safety Set
SCIT: subcutaneous immunotherapy
SOC: System Organ Class
TEAE: treatment-emergent adverse event
TU: therapeutic units
WAO: World Allergy Organization
